# Development of the Visual Analysis of Form and Contour

**DOI:** 10.3390/children12081005

**Published:** 2025-07-30

**Authors:** Clay Mash, Lauren M. Henry, Marc H. Bornstein

**Affiliations:** 1 National Institutes of Health, United States Department of Health and Human Services, Bethesda, MD 20852, USA; 2 Eunice Kennedy Shriver National Institute of Child Health and Human Development, National Institutes of Health, United States Department of Health and Human Services, Bethesda, MD 20892, USA; 3 National Institute of Mental Health, National Institutes of Health, United States Department of Health and Human Services, Bethesda, MD 20852, USA; 4 Institute for Fiscal Studies, London WC1E 7AE, UK; 5 UNICEF, New York City, NY 10017, USA

**Keywords:** infancy, visual perception, perceptual development, illusory contours

## Abstract

Background/Objectives: A common approach to investigating visual form processing is through studying responses to visual stimuli that comprise illusory contours. Such stimuli induce contours where none exist physically and thus reveal the constructive nature of visual perception and the conditions that engender it. The present work used IC stimuli to study the development of visual form detection and extraction in infants and adults. Methods: Infant and adult participants viewed square stimulus forms with either real or illusory contours, while their looking behavior was measured with an eye tracker. Fixations of the stimuli were coded by region, distinguishing between the contours of the forms and within the forms themselves. Fixations were summed by region, and fixations on forms were interpreted to index the detection of coherent, whole forms. Fixations on contours (real and illusory) were interpreted to index the extraction of form edges. Results: Total form fixations differed by age. For real contours, fixations by infants exceeded those by adults; when contours were illusory, adult fixations were greater than those of infants. Contour fixations were similar between ages. Infants and adults both looked more at contours when illusory than when real. Conclusions: Together, the results provide new conclusions about change and continuity in the visual analysis of form and contour. The results suggest that the visual detection and binding of simple form structure appears to develop between infancy and adulthood. However, the exploration of contours that support the extraction of form contours from backgrounds appears to change little between infancy and adulthood.

## 1. Introduction

A central question about the development of visual perception is how vision transcends the stimulation available to retinal surfaces (e.g., [1]). The richness of perceptual experience suggests that constructive processes intertwine with current sensations in visual processing. This notion gains particular relevance in relation to challenges like occlusion and disconnected surfaces that are ubiquitous in the visual environment and which engender incomplete, two-dimensional retinal projections. Astonishingly, the visual system adapts to this variation and adeptly fills in these gaps [2].

**Figure 1:**
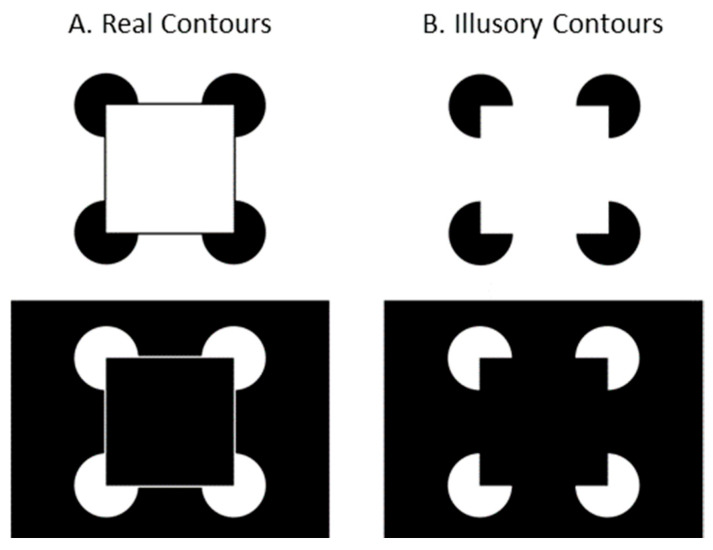
Real (**A**) and illusory (**B**) contour stimulus patterns used in the experiment.

Illusory contours (ICs), a class of stimuli inducing perception of complete contours from incomplete ones, have emerged as a cornerstone in research to understand contour integration processes. The Kanizsa square [3], one of the most notable examples of ICs, consists of figures arranged to suggest the presence of a square (see Figure 1B). Instead of perceiving the image as a coincidental arrangement of four elements in different orientations, viewers typically perceive a square positioned in front of four circular disks. The perception of ICs in the absence of physical presence suggests that the human visual system might represent the form contours explicitly. Importantly, ICs demonstrate that our visual system does not merely process a retinal image but actively constructs perceptual features.

Along with a large literature on adults’ IC perception (e.g., [4,5]), developmental aspects of performance and the processes involved in IC perception have also been explored. As early as 3 to 4 months of age, infants have demonstrated the ability to detect and perceive ICs [6]. Much of the research on early IC perception implemented preferential looking tasks and habituation. As one example, Ghim [7] prepared stimuli in which elements were either positioned and oriented to induce ICs or in the same position but with 2 of 4 elements rotated in different orientations so as not to induce ICs (non-IC). Ghim familiarized infants to one stimulus of either type (IC or non-IC) by presenting the stimulus repeatedly over several trials while the duration of infant looking at the stimulus was measured. When infants were familiarized to an IC stimulus and tested with a non-IC stimulus, they looked longer at the non-IC stimulus. When infants were familiarized to a non-IC stimulus and tested with a different non-IC stimulus (different elements rotated), they did not look longer at the test stimulus. Use of this design and other related paradigms has revealed clear infant sensitivity to ICs (see [8] for review of infant research). These findings are not an artifact of static Kanizsa stimuli in particular as other researchers have documented sensitivity of infants to other IC structural formats [8,9], as well as under movement conditions [10,11]. A substantial and growing body of literature now supports the conclusion that the visual construction of form contours is not only a core capacity of mature perception but is also present and apparent very early in life.

Despite this progress, important questions remain about the course of development in the construction of forms that IC sensitivity reflects, and what visual behaviors relate to contour perception and its development. In the current study, we use Kanizsa stimuli and visual fixation measurement to study the development of form construction and extraction by examining perceptual equivalence between real and visually induced form contours. Specifically, we examine whether real and illusory contours differentially affect form fixations and contour fixations. Our research follows two well-grounded assumptions in the extant literature.

First, fixations of a visual form within its outer contours are facilitated by, and thus reflect, how well the form is perceptually organized, bound into a whole, and extracted from its background [12,13,14]. Einhäuser, Spain, and Perona [15], for example, analyzed stimulus scene images to map the images’ feature salience and object presence. Adults’ eye movements over those stimulus images corresponded more with the positions of recognizable objects in the scenes than with other salient features in the scenes, corresponding to what participants remembered about the scenes subsequently. Fixations picked out objects even when the objects were competing with other salient feature variations.

A second assumption is that fixations of contour locations reveal exploration of scene content and the identification of surface discontinuities that could correspond to objects. Infants are sensitive to cues that adults use to organize complex displays, such as gestalt relations [16,17], and preattentive cues to object presence such as rapid detection of discrepant elements in large arrays [18,19]. Also, infants scan displays to visually construct objects when those displays are partly occluded [20]. These and other findings support the premise that exploratory scanning is both analytic and constructive across ages in at least some task contexts. Such exploration supports the extraction of object edges from their surrounding [21,22].

Each of these aspects of visual analysis—form fixation and contour fixation—are examined separately in the present experiment. Infants and adults viewed square stimulus forms having either real or illusory contours while their looking behavior was measured with an eye tracker. Fixations of the stimuli were coded by region, distinguishing between the contours of the forms and within the forms themselves. Fixations were summed by region, and fixations on forms were interpreted to index the detection of coherent, whole forms. Fixations on contours (real and illusory) were interpreted to index the extraction of forms’ edges. Performance is compared between age groups to examine stability and change in form detection and contour extraction.

## 2. Materials and Methods

In total, 16 infants (M age = 156.3 days, range = 26.7, SD = 8.9; 9 females) and 12 adults (M age = 26.1 years, range = 18.3, SD = 6.0; 9 females) participated in the current study, recruited on the basis of age and parent interest. An additional 7 infants began the procedure but were not included due to fussiness, inability to calibrate equipment adequately, or equipment failure. Sample sizes were determined by reference to previous related research (e.g., [23,24]). Data collection ceased when planned sample sizes were reached. Of note, the attrition rate is comparable to other published eye tracking studies [25]. Infants were recruited through the use of purchased mailing lists of newborns in a suburban metropolitan area. Infants represented families of middle to high socioeconomic status. All infants were term and healthy at birth and at the time of testing. Mothers of infants completed informed consent forms prior to data collection. Adults were recruited from institutional staff.

Stimuli were Kanizsa squares composed of four circular inducing elements (see Figure 1B) and Kanizsa forms modified with the addition of real contours (Figure 1A). Both patterns were represented with white inducing elements on black backgrounds, and black inducers on white backgrounds. The patterns subtended 27° on the diagonal between outer edges of the inducing elements.

An Applied Science Laboratories (ASL; Bedford, MA, USA) Model 504 eye tracking system was used to measure eye fixations for each stimulus image. The system uses infrared corneal reflection to record fixation coordinates on the stimulus plane continuously at 60 Hz. An ASL video head tracker was used to correct camera angles for spontaneous head movements that exceeded the frame limits of the optical tracking. Signals from the head tracker were integrated with the eye camera control unit and used to guide the camera’s pan/tilt motors when corneal reflections were lost. Eye movement recording was synchronized with stimulus presentation using GazeTracker_TM_ v1.0 (EyeResponse Technologies, Charlottesville, VA, USA) software that was running on a second microprocessor. The stimulus presentation microprocessor was linked to the system’s primary CPU by their respective serial ports. Stimuli were presented on a 48 cm diagonal Panasonic monitor, and the eye camera was situated beneath the stimulus monitor.

Infants were seated in an infant chair, and adults were seated in a regular chair, ~60 cm from the eye camera and stimulus monitor. The eye tracking system was calibrated for each participant individually by presenting a rotating red plus sign (1.27°) in the upper left and lower right corners of an otherwise uniform white field. When participants were judged to be fixating the targets, the known locations of those targets were mapped onto the corneal reflections for each participant using the ASL calibration procedure.

**Figure 2:**
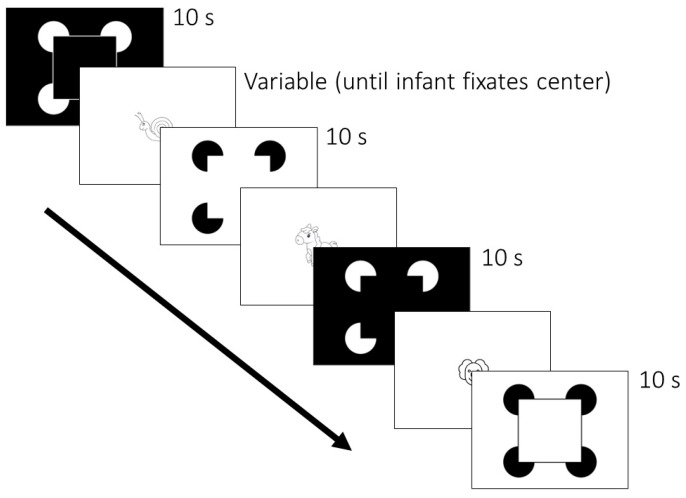
Sequence example of four 10 s trials depicting each stimulus once. Sixteen trials were presented in counterbalanced random order. Trials began when infants were judged to be looking at the center of display.

Participants were shown 4 stimuli over 16 trials in 4 randomized and counterbalanced orders (Figure 2). Each of the 4 stimuli is pictured in Figure 1. Stimuli were presented for 10 s each. Between trials, animated cartoon figures were presented to maintain each infant’s attention to the stimulus monitor. The inter-trial cartoon figures averaged 5° × 5° in visual angle, and a different one was used after each trial. Trials were initiated by a key press when the infant or adult participant was judged to be looking toward the display.

**Figure 3:**
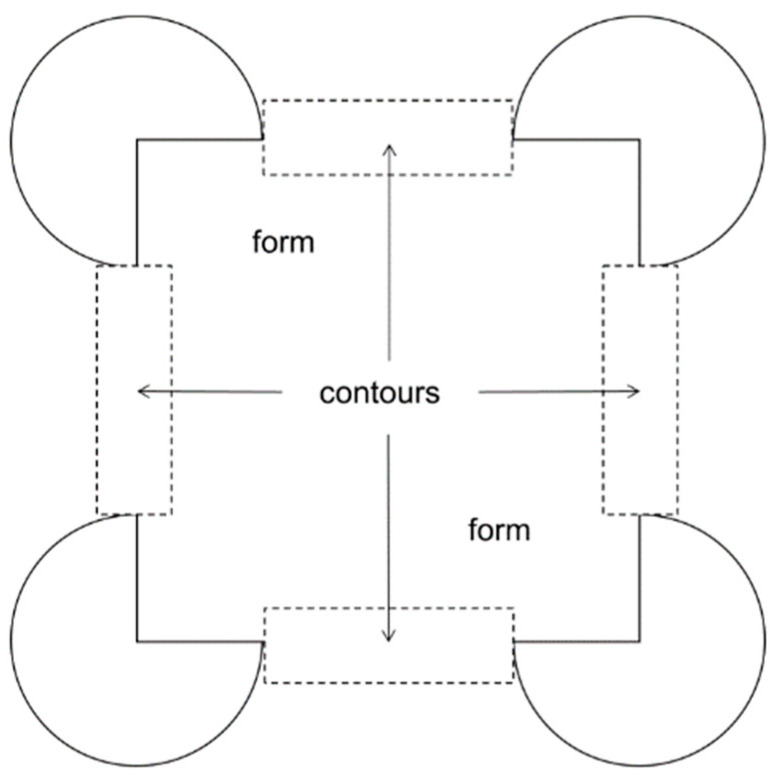
Regions of interest in coding fixations.

To analyze data from the eye tracking system, fixations of 200 ms and more were plotted directly on the stimulus image for each trial for each participant using the GazeTracker software package. The scan plots were then analyzed by coders blind to the conditions, parameters, and hypotheses of the experiment. Fixations were classified as “form” if they fell on any other portion of the pattern excluding the inducing elements, and as “contour” if they fell within 1° of the (real or illusory) contour boundary beyond the inducing elements surrounding the four corners. See Figure 3 for a depiction of the form and contour regions of interest for coding fixations.

The analysis options in GazeTracker were set to include gaze point changes of less than 1° of visual angle as part of the same fixation. Any gaze point changes greater than 1° of visual angle were coded as a shift between consecutive fixations anywhere on the target. Visual analysis of the stimulus images was defined as the number of fixations tallied by region (form or contour) for each trial. To control for individual differences in overall fixation shift frequency, fixations were converted to proportions by dividing the contour and form totals into the total number fixations of the entire stimulus pattern for each trial and averaging over trials. Fixations of the stimulus screen outside of the coded regions of interest were not further analyzed. Twenty-five percent of the sessions were coded by a second independent observer, and coders’ ratings coincided for 100% of the individual trials.

## 3. Results

The participant sampling and analytic design reflect conventional practice across infant eye tracking studies, and no a priori power analysis was conducted. The analyses were designed to compare form fixations and contour fixations between contour conditions and age groups. Accordingly, form fixation proportions and contour fixation proportions were analyzed in separate 2 (real vs. illusory contour conditions, within groups) × 2 (infants vs. adults, between groups) analyses of variance (ANOVA).

Effect sizes were interpreted with η_p_^2^ (small: <0.01, medium: >0.06, large: >0.14). Because variance in proportions of fixations on forms bounded by illusory contours was not homogeneous (Levene’s test F = 7.282, *p* = 0.012), proportions were log transformed prior to analysis to normalize their distribution. Preliminary analyses revealed no effects of sex or trial order on fixation totals, and subsequent analyses collapsed across these variables.

**Figure 4:**
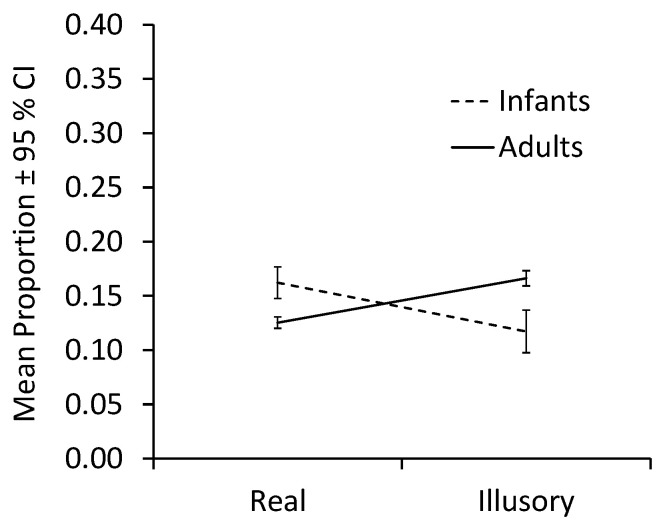
Mean proportions of fixations on the form by contour condition and age group.

Figure 4 plots mean proportions collapsed across infants and adults. The analysis of fixations of the form revealed a significant interaction between Contour condition and Age group, F(1,26) = 7.49, *p* = 0.011, η_p_^2^ = 0.22. Neither the main effect of condition, F(1,26) = 0.37, *p* = 0.55, nor age group, F(1,26) = 3.73, *p* = 0.07, was significant.

Because sample sizes were unequal between groups, we conducted bootstrap analyses to test the simple effects using a 95% CI and resampling 2000 times. When contours were illusory, proportions of adult fixations on the form were greater than proportions of infant fixations on the form, MD = −0.493, 95% CI [−0.857, −0.202], Welch’s t(16.205) = −2.908, *p* = 0.041, d = −0.966. With real contours, infant fixations on the form were greater than adult fixations on the form, MD = 0.234, 95% CI [0.098, 0.374], Welch’s t(22.003) = 3.249, *p* = 0.005, d = 1.125. For contrasts between contour conditions within age groups, fixations by infants did not differ significantly, MD = 0.444, 95% CI [0.017, 0.881], t(15) = 1.988, *p* = 0.088, d = 0.497. Adults, however, fixated more on forms bound by illusory contours than those bound by real contours, MD = −0.282, 95% CI [−0.411, −0.151], t(11) = −4.242, *p* = 0.004, d = −1.225.

Thus, while infants fixated forms bounded by real contours more than adults did, the adults fixated forms bounded by illusory contours more than infants did.

**Figure 5:**
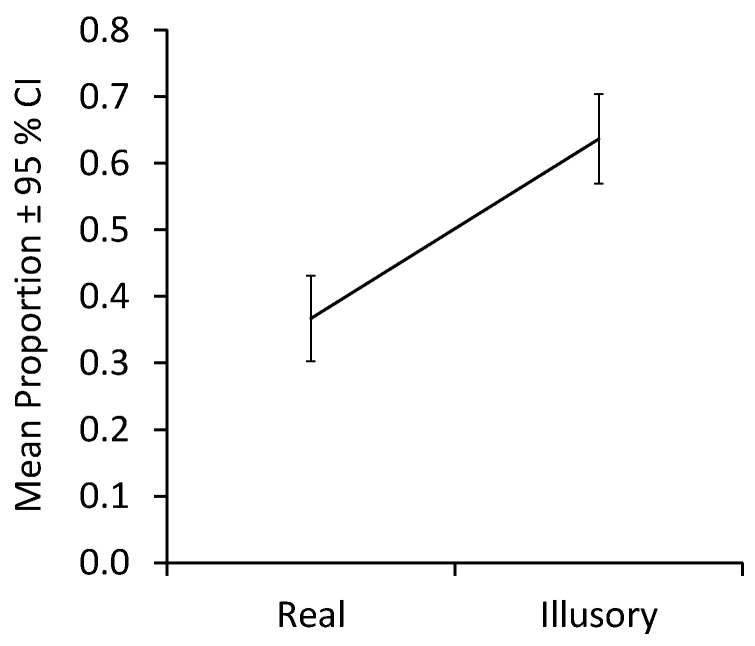
Mean proportions of fixations on the contour regions by contour condition collapsed across infants and adults.

The analysis of contour fixations revealed a large main effect of contour condition, F(1,26) = 9.95, *p* = 0.004, η_p_^2^ = 0.28, with more contour fixations across age groups occurring in the absence of real contours than in their presence. Figure 5 plots means collapsed across infants and adults. Neither the effect of age group, F(1,26) = 1.59, *p* = 0.22, nor the interaction of Contour condition × Group, F(1,26) = 1.49, *p* = 0.23, was significant. Analyses of simple effects revealed no difference between age groups for regions with either real contours, MD = −0.439, 95% CI [−1.117, 0.098], Welch’s t(19.685) = −1.419, *p* = 0.215, d = −0.483, or for illusory ones, MD = 0.096, 95% CI [−0.125, 0.313], Welch’s t(25.912) = 0.875, *p* = 0.394, d = 0.323. Both infants (MD = −2.10, 95% CI [−1.723, −0.340], t(15) = −2.728, *p* = 0.044, d = −0.682) and adults (MD = −0.425, 95% CI [−0.790, −0.569], t(11) = −2.265, *p* = 0.050, d = −0.654) fixated more on regions with illusory contours than on those with real ones.

In sum, both infants and adults fixated on contour regions more when they were illusory than when they were real.

## 4. Discussion

Forms and contours constitute foundational elements of visual perception, acting as the cornerstones for constructing our understanding of the visual world. Under most circumstances, the presence of object surfaces is defined by enclosed contours marked by contiguous changes in luminance, texture, or color. In the primary visual cortex, simple and complex cells play pivotal roles in extracting local contour details, channeling the information through a hierarchy of visual processing stages to facilitate the perception of objects and surfaces (e.g., [26]). Yet, the human visual system excels at detecting visual contours even when local image information is not replete with complete luminance, texture, or color cues as examined here with IC stimuli. These stimuli serve as powerful tools for studying mechanisms of contour and surface perception as well as constructive visual perception. Here, we explored the perceptual equivalence of real and visually induced form contours in infancy and adulthood. To do so, we examined visual scanning between forms defined by ICs and similar forms defined by real contours.

On the basis of performance in the task used in this work, visual analysis of the form itself differed between these two age groups. When contours were real, infants pro fixated on the form more than adults. By contrast, when contours had to be induced adults fixated more on the form than did infants. On the assumption that more fixations of the form reveal a more organized and coherent visual structure, form binding appears to depend more on real contours for infants than it does for adults. Bulf, Valenza, and Simion [27] reported findings consistent with these. They used a visual search task with 6-month-old infants and with adults. The task involved an embedded Kanizsa square having illusory or real contours in an array of several inducing elements that were not arranged or aligned to induce contours. Adults’ fixations were drawn to the form rapidly and with the same latency over differing numbers of distractors. This finding suggests that the forms were detected preattentively by adults and were so for both real and illusory figures. By contrast, infants appeared to detect only the real figures preattentively. Bulf et al. [27] concluded that the performance difference they observed was likely due to a limitation among immature perceivers to perceptually bind the structure of the illusory figures, a finding consistent with that presented here. The basis of the age difference that this work revealed may be a product of many changing factors and processing stages ranging from contrast sensitivity to perceptual learning—including the physiological bases of each—and further work will be necessary to identify them.

In the present task, we also found that infants and adults alike fixated more on contours when they were illusory than when real. This pattern is plausibly interpreted in terms of greater exploratory analysis in efforts to extract coherent pattern information from a stimulus when information is not directly specified by the stimulus structure. Notably, this strategy did not appear to differ between early infancy and young adulthood. Exploration is a foundation of visual perception and, as such, may be developmentally fundamental and constant [21]. A key element of Gibson’s theory of perceptual learning and development is that perceivers actively explore to gather information to guide behavior, and that visual exploration is evident from the earliest days serving the same functional purpose throughout life [28]. The present findings corroborate that position with the observation of equivalent levels of visual exploration between infants and adults.

A limitation of this work is that we examined only a single form with the present stimulus set which may constrain the generalizability of the results. Tests with a broader array of differing forms are called for in further research. Another potential limitation of the work is that the sample size in both age groups was small. However, the sampling is in line with other studies in this area, and proved statistically adequate for testing the phenomena of interest. Participating in attention-demanding tasks like the one used in the present study can be taxing, particularly for infants. For that reason, testing more participants than is necessary for addressing the planned research question carries an undue burden. Additionally, the pattern of results reduces concern about statistical power limitations. The analysis of fixations detected relevant simple effects, and mean proportion fixation time on contour regions was so similar between age groups that an unreasonably large sample would be required to detect any possible differences. Nevertheless, replication of the current findings as well as additional research addressing these limitations are both warranted. Follow-up work comparing form fixations between additional age groups will also be necessary to identify the age course of emergence and change in form biding. Nayar and colleagues [29], for example, reported substantial developmental changes in the processing of illusory contours between 4 and 7 years of age.

In conclusion, the ability to bind the results of visual exploration into coherent forms appears to develop between infancy and early adulthood. This finding corroborates previous findings of form perception as a developmental product [17] while discounting early changes in visual exploration as a likely explanation of that development. Infants and adults employed similar visual strategies in analyzing edges of real and illusory contours, revealing very little difference with age. This continuity reinforces existing characterizations of humans as active explorers of their visual environments not only at maturity but also from their earliest days.

## Data Availability

Due to consent restrictions, data are available only upon request to the corresponding author.
